# Celebrating the 10th Anniversary of *ACS Central Science*

**DOI:** 10.1021/acscentsci.3c01604

**Published:** 2024-01-24

**Authors:** Carolyn R. Bertozzi, Kirk S. Schanze

*ACS Central Science* is approaching a milestone—our 10th year of publishing leading-edge research across the chemical sciences and allied fields, and we could not be more proud! Not only has the journal solidified its stature as a flagship within the American Chemical Society portfolio, we remain the epicenter of innovation in scientific publishing. *ACS Central Science* was the first fully open access ACS journal, an experiment that was so successful it set the stage for an expansion to the dozen hosted by ACS Publications today. *ACS Central Science* still holds a special place at ACS as the only *Diamond open access* journal in the 80+ journal portfolio. We also launched ACS’s first transparent peer review system in collaboration with *The Journal of Physical Chemistry Letters*, unlocking the value of reviewers’ hard work and shedding light on the peer review process for the benefit of trainees. Our success reflects a talented team of Senior Editorial Board members, ACS staff, and most importantly, authors, reviewers, and readers dedicated to communicating science across disciplines and impacting the world.

**Figure 1:**
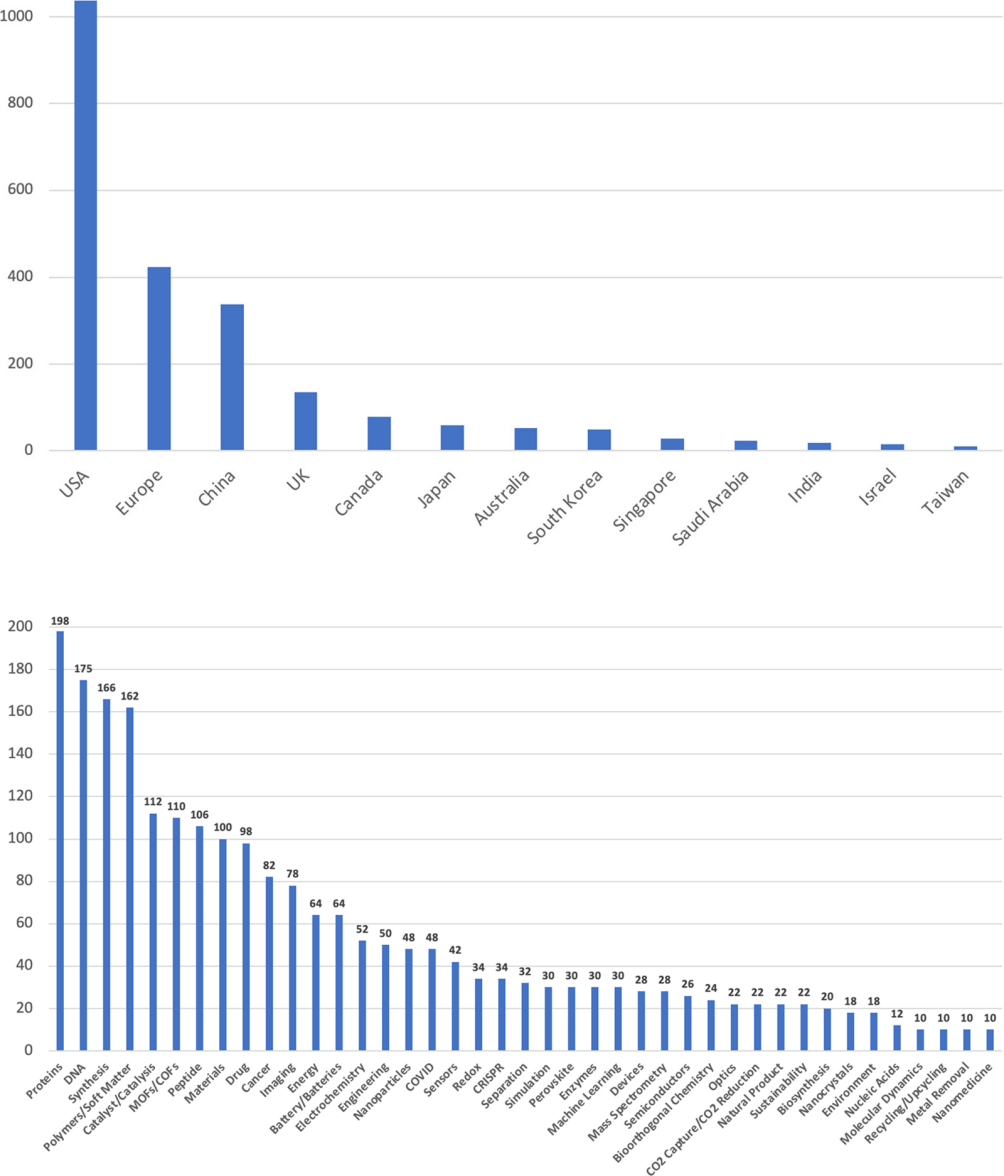
(Top) Geographic distribution of papers published in *ACS Central* Science. (Bottom) Topic analysis of published items appearing in *ACS Central Science* as of 12/10/2023.

In celebration, we share here a retrospective look at the journal’s trajectory over the past decade and project forward to where things may go in the coming decade (noting that it is much easier to look backward than to predict the future!). Figure 1 highlights the regional and topical distribution of articles published in *ACS Central Science* during the past decade. The global reach of the journal is evident in the regional distribution in Figure 1a, with the authors from the US, Europe, China, and the UK contributing the largest number of papers, but with substantial numbers coming from authors across the globe. An analysis of the topics that frequently appear in the journal is provided in Figure 1b, which shows a topical breakdown of the published papers. While this analysis is rather generic, close inspection reveals some trends. First, the most frequent topics appearing are related to biochemistry and chemical biology (e.g., proteins, DNA, peptide, drug, cancer, biorthogonal chemistry, and enzymes). Synthesis and catalysis are also frequent keywords. Porous materials, including metal organic frameworks (MOFs) and covalent organic frameworks (COFs) are also frequently featured in the journal. Other topics that are relevant to materials chemistry and materials science that appear in Figure 1b include batteries, semiconductors, and perovskite materials.

During the past decade, several key areas of chemical science have been prominent in the articles published in *ACS Central Science*. These areas are highlighted by the following articles that have been either highly cited, highly downloaded, or noted by the editors. The field of artificial intelligence (AI) applied to problems in the chemical sciences has been prominently featured in *ACS Central Science*. The most highly cited article in the journal to date is entitled, “Automatic Chemical Design Using a Data-Driven Continuous Representation of Molecules” authored by Aspuru-Guzik and co-workers. This article describes the development of exploring chemical space using continuous molecular encodings. Other highly cited AI focused articles include, “Generating Focused Molecule Libraries for Drug Discovery with Recurrent Neural Networks” by Waller and coauthors, “Prediction of Organic Reaction Outcomes Using Machine Learning” by Green and co-workers, and “Molecular Transformer: A Model for Uncertainty-Calibrated Chemical Reaction Prediction” by Schwaller and co-workers.

Given the significance of the COVID-19 pandemic and the impact on the research community, it is not surprising that there are many impactful papers that have appeared in *ACS Central Science* on the subject of COVID-19-related research and drug and vaccine development since 2020. Prominent among the research articles are “Beyond Shielding: The Roles of Glycans in the SARS-CoV-2 Spike Protein” by Amaro and coauthors and “The SARS-COV-2 Spike Protein Binds Sialic Acids and Enables Rapid Detection in a Lateral Flow Point of Care Diagnostic Device” by Gibson and coauthors. An In Focus article published in May of 2020 has been featured as one of the most cited and downloaded papers published to date in the journal, “Research and Development on Therapeutic Agents and Vaccines for COVID-19 and Related Human Coronavirus Diseases”. This piece has been viewed >350 000 times and serves as an outstanding example of the benefit of open access making science available to a broad readership.

As noted above, the largest cohort of articles published in the journal over the past decade are in the fields of biochemistry and chemical biology. Among the most-cited biochemistry articles is the paper “On the Mechanism of Cytoprotection by Ferrostatin-1 and Liproxstatin-1 and the Role of Lipid Peroxidation in Ferroptotic Cell Death” by Pratt and coauthors. This paper probes the mechanism of cytoprotection by the ferroptosis inhibitors ferrostatin-1 and liproxstatin-1. Another noteworthy article that has been frequently cited and is on the topic of ferroptosis is, “GTP Cyclohydrolase 1/Tetrahydrobiopterin Counteract Ferroptosis through Lipid Remodeling” by Stockwell and coauthors. The area of chemical biology is well represented in the journal with noteworthy articles including, “Site-Selective and Rewritable Labeling of DNA through Enzymatic, Reversible, and Click Chemistries” by Neely and co-workers, as well as “CYP450 Enzymes Effect Oxygen-Dependent Reduction of Azide-Based Fluorogenic Dyes” by Conway and coauthors.

Not surprisingly, the field of materials science, with applications to catalysis, energy storage, and energy conversion, have been featured in a number of prominent articles in *ACS Central Science*. A particularly noteworthy article in the field of electrochemical energy storage is “A Highly Reversible Room-Temperature Sodium Metal Anode” by Cui and co-workers. This article reports the use of NaPF_6_/glyme in the electrolyte to mitigate dendrite formation at a sodium anode during electrochemical cycling. An article on a related topic regarding dendrite and pit formation in lithium anodes is “Dendrites and Pits: Untangling the Complex Behavior of Lithium Metal Anodes through Operando Video Microscopy” by Dasgupta and coauthors. Exemplary articles in the field of materials for electrocatalysis include, “In Situ Electrochemical Oxidation Tuning of Transition Metal Disulfides to Oxides for Enhanced Water Oxidation” also by Cui’s group and “A Direct Grain-Boundary-Activity Correlation for CO Electroreduction on Cu Nanoparticles” by Kanan and coauthors.

Porous materials have also been active topics in the journal, and among these is the highly downloaded outlook entitled, “Metal–Organic Frameworks for Water Harvesting from Air, Anywhere, Anytime” by Xu and Yaghi. This article provides a general-reader style discussion of the application of MOFs to water harvesting in all types of climates. Two primary research articles reporting on specific MOF materials for water harvesting include “Record Atmospheric Fresh Water Capture and Heat Transfer with a Material Operating at the Water Uptake Reversibility Limit” by Dincă and coauthors and “Rapid Cycling and Exceptional Yield in a Metal-Organic Framework Water Harvester” also from the Yagi group. Another nice study that brings together the fields of porous and electronic materials is reported in the article “Single Crystals of Electrically Conductive Two-Dimensional Metal–Organic Frameworks: Structural and Electrical Transport Properties” by Dincă’s laboratory.

Finally (and not least important), is the field of synthesis and catalysis. These areas have also been prominent in the journal during the past decade. A noteworthy article in the field of organic catalysis and synthesis is “Remote Meta-C–H Activation Using a Pyridine-Based Template: Achieving Site-Selectivity via the Recognition of Distance and Geometry” by Jin-Quan Yu and coauthors, which describes a strategy of using pyridine to direct remote C–H activation by Pd-catalysis. Also of note is “Chemoselective Aliphatic C–H Bond Oxidation Enabled by Polarity Reversal”, by Bietti and coauthors.

During its first decade of publishing, *ACS Central Science* has become an established journal in the field of the chemical sciences. However, looking forward we see that there are areas where the journal could benefit from further development. First, more than 60% of the articles published to date in the journal are submitted by US authors. It is hoped that in the future the geographical distribution for papers published in the journal could be evened out, with a greater fraction of the publications coming from diverse regions. We strongly encourage authors from around the globe who are working in the chemical sciences to consider submitting their impactful papers for publication in *ACS Central Science*. Second, the journal could benefit by diversification of the topical areas represented, and it is hoped that over the coming few years the editors can work with the author community toward this goal.

Finally, in closing we wish to acknowledge the efforts of the authors, reviewers, editorial advisory board, the editors, and ACS staff who have been strong and continuous supporters of the journal during its first 10 years of publishing open access articles in the field of chemical sciences. We are especially indebted to Prof. Christopher Chang, who has served as one of the founding Senior Editors and then Deputy Editor. We wish Prof. Chang well as he moves to his new position as Editor in Chief of *Accounts of Chemical Research.* His tireless efforts in support of the journal have been immensely appreciated.

